# Microbiological and SEM-EDS Evaluation of Titanium Surfaces Exposed to Periodontal Gel: In Vitro Study

**DOI:** 10.3390/ma12091448

**Published:** 2019-05-04

**Authors:** Sara Bernardi, Serena Bianchi, Anna Rita Tomei, Maria Adelaide Continenza, Guido Macchiarelli

**Affiliations:** 1 Department of Life, Health and Environmental Sciences, University of L’Aquila, 67100 L’Aquila, Italy; serena.bianchi@univaq.it (S.B.); mariaadelaide.continenza@cc.univaq.it (M.A.C.); gmacchiarelli@univaq.it (G.M.); 2 “San Salvatore” City Hospital, 67100 L’Aquila, Italy; artomei@asl1abruzzo.it

**Keywords:** doxycycline, implant properties, peri-implantitis

## Abstract

Inflammatory diseases affecting the soft and hard tissues surrounding an implant represent a new challenge in contemporary implant dentistry. Among several methods proposed for the decontamination of titanium surfaces, the administration of topical 14% doxycycline gel seems to be a reliable option. In the present study, we evaluated the microbial effect of 14% doxycycline gel applied on titanium surfaces and exposed to human salivary microbes in anaerobic conditions. We also examined the composition of the exposed surfaces to assess the safe use of periodontal gel on titanium surfaces. Six anatase and six type 5 alloy titanium surfaces were used and divided into two groups: The test group and the positive control group. Both were cultured with human salivary samples in anaerobic conditions. On the test groups, 240 mg of periodontal gel was applied. The microbial assessment was performed with a colony-forming unit (CFU) count and matrix-assisted laser desorption ionization-time of flight (MALDI-TOF) to identify the species. The surface integrity was assessed using scanning electron microscopy-energy dispersive X-ray spectrometry (SEM-EDS). The results demonstrated the microbial efficacy of the 14% doxycycline periodontal gel and its safe use on titanium surfaces. However, the SEM observations revealed the permanence of the gel on the titanium surfaces due to the physical composition of the gel. This permanence needs to be further investigated in vivo and a final polishing protocol on the titanium surface is recommended.

## 1. Introduction

Implantology is the branch of dentistry that was specifically developed with the aim of restoring a tooth that has been extracted due to disruptive caries or periodontitis, or that was missing due to agenesis [1,2,3,4]. The inner nature of this specialty leads to the research and improvement of materials able to replace the dental root, to integrate into the alveolar bone tissue, and to functionally support the prosthetic structure [2]. 

Titanium and its derived alloys were found to be the most suitable to be used in implant dentistry. Indeed, this element presents biocompatibility as well as corrosion and mechanical resistance properties [5]. The biocompatibility and resistance to corrosion are due to the formation of a film consisting of amorphous titanium dioxide (TiO_2_) on the surface of the titanium [5,6]. Moreover, since implant fixtures made of titanium represent a direct connection between the oral environment and the alveolar bone, the control of the microbial biofilm, which physiologically inhabits the oral cavity, is crucial for the success of the implant therapy [7]. 

Indeed, if at the beginning implant therapy appeared as the perfect solution for tooth replacement, long-term studies have proved that implants could also be involved in inflammation and infection, which result in a high risk of losing the fixture. As a consequence, a new oral disease has shown up—peri-implantitis [8,9,10]. 

According to the new classification scheme from the 2017 world workshop of the American Academy of Periodontology and the European Federation of Periodontology, the healthy status of peri-implant tissues is characterized by “an absence of visual signs of inflammation and bleeding on probing” [11]. On the other hand, in the case of disease, two conditions are identified and classified: Peri-implant mucositis and peri-implantitis. The former presents bleeding on probing, with inflammatory characteristics, is reversible and plaque-dependent. The latter, which is also plaque-dependent, presents inflammation of peri-implant mucosa and the loss of surrounding bone tissue [11]. 

Several studies have reported how the microbial population of the biofilm characterizing peri-implantitis is mainly composed of *Aggregatibacter actinomycetemcomitans*, *Porphyromonas gingivalis*, *Prevotella intermedia*, and *Treponema denticola* [12]. Since this harmful biofilm is the primary cause of peri-implantitis, different protocol treatments were studied and introduced to remove or at least decrease the microbial load. These treatments are mechanical, such as manual debridement, ultrasonic debridement, air-abrasive device, and laser decontamination [13,14,15]. The mechanical treatments, however, are limited, and when the rough portion of the implant surface is involved, the situation gets complicated [16,17,18]. 

Hence, the combination of the mechanical and local application of antibiotics and/or antiseptics has been found to be one of the most promising strategies to address peri-implantitis [19]. 

Among the available antibiotic molecules, tetracyclines were shown to be efficient, especially against subgingival microorganisms [20]. Tetracyclines, indeed, present an inhibiting action on destructive enzymes such as matrix metalloproteinases and collagenases. Besides, doxycycline stimulates osteoblasts by promoting cell maturation and differentiation and has a resulting healing effect on bone tissue [20]. 

Some studies assessed the efficacy of the administration of 14% doxycycline gel as a non-surgical adjuvant in the treatment of periodontitis [19]. Recently, two studies showed promising antimicrobial results using 14% doxycycline gel on implant surfaces [19,20]. However, neither of these studies evaluated the combined effect that the gel (which has to remain on the surface for 12 days and biodegrades 50 days after application) and the salivary microbes has on the smooth titanium surface of the implants. 

The aim of this study is to evaluate not only the microbial effect of 14% doxycycline gel applied on titanium surfaces that are exposed to human salivary microbes in anaerobic conditions, but also to examine the composition of the exposed surfaces to assess the safe use of the periodontal gel on titanium surfaces. 

## 2. Materials and Methods 

### 2.1. Samples and Sampling Procedure

Fourteen healing screws kindly provided by Maco Dental Care (Maco International S.A.S., Salerno, Italy) were used. Seven screw samples presented a surface of titanium alloy grade 5 (Ti AL6V4 ELI), and seven had a surface of anodized titanium. The difference between the two surfaces consists of the distribution on the surface of dioxide titanium and in their chemical composition: The titanium alloy grade 5 has 6% aluminum, 4% vanadium, and the rest titanium. The anodized titanium surface has also small percentages of phosphorous and a higher concentration of oxygen compared to the the grade 5 titanium alloy. 

The microorganisms that we used for the experiment were derived from a saliva sample.

The source of saliva was a 60 years old male volunteer suffering from chronic periodontitis. A dentist assessed the general and oral health status of the volunteer.

The criteria inclusions for the recruitment of the volunteer were as follows:

- Good oral hygiene level;
- Periodontal Screening and Recording Index: 2;
- Good general health according to the American Society of Anesthesiologists’ physical classification system.

Ten milliliters of saliva were obtained early in the morning before tooth brushing. 

The healing screws were divided into six groups:

- Group A, consisting of three healing screws in titanium anatase seeded with the salivary microorganisms and without the application of the doxycycline gel;
- Group B, consisting of three healing screws in titanium anatase seeded with the salivary microorganisms and with the application of the doxycycline gel;
- Group C, consisting of three healing screws in grade 5titanium alloy seeded with the salivary microorganisms and without the application of the doxycycline gel;
- Group D, consisting of three healing screws in titanium alloy grade 5 seeded with the salivary microorganisms and with the application of the doxycycline gel on the surface;
- Group E, consisting of one healing screw in grade 5 titanium alloy not exposed to any procedure, and used as a negative control in the SEM-energy dispersive X-ray spectrometry (EDS) procedure;
- Group F, consisting of one healing screw in anatase not exposed to any procedure, and used as a negative control in the SEM-EDS analysis.

Kulzer GmbH, Hanau, Germany kindly provided the 14% doxycycline gel (Ligosan ^®^, Kulzer GmbH, Hanau, Germany).

The chemical composition of the 14% doxycicline gel includes a hydrogel functioning as a carrier, and consists of polyglycolide and macrogol-DL-lactide at high and low viscosities.

### 2.2. Microbiological Procedure

Each group (A, B, C, and D) was cultured with 300 µL of saliva in 100 mL of broth medium (Anaerobe Basal Broth, Oxoid, Thermo Fisher, Rodano, Italy) in anaerobic conditions for seven days.

In groups A and C, the quantity of Ligosan^®^ administrated was 240 mg for each group with the ratio of 240:3. 

After culturing the broth medium, two healing screws from each group were submerged in phosphate buffered saline (PBS) solution and then sonicated for 10 min at 60 Hz and 100 W. Afterward, the PBS solution arising from that was seeded in agar blood medium for anaerobes and incubated for three days in anaerobic conditions. 

Then, colony-forming units (CFUs) were eye-counted and recorded. Finally, we proceeded with the identification of the microbial strains using a matrix-assisted laser desorption ionization-time of flight (MALDI-TOF, Bruker, Billerica, MA, USA) facility. 

The remaining healing screws from each group were fixed in glutaraldehyde 2% for the scanning electron microscopy and energy dispersive X-ray spectrometry analysis.

### 2.3. Scanning Electron Microscopy and Energy Dispersive X-ray Spectrometry (SEM-EDS) Analyses 

The protocol of the preparation of the samples for SEM observation was followed as described before. Briefly, after the fixation of the screws, they were dehydrated in an ascending series of alcohols (50%, 75%, 95%, 100%), allowed to dry on absorbent paper for 48 h, and observed with the scanning electron microscope (GEMINI_SEM, Zeiss, Germany). The surfaces were randomly observed different locations at different degrees of magnification in secondary electrons (SE) mode [21]. The used parameters were acceleration voltage (AV) 7.00 kV, spot size 20 μm, and working distance between 13.6 and 14.1 mm. The healing screws were sonicated in 70% alcoholic solution with distilled water to perform EDS analyses after the SEM morphological observations. The above procedure was necessary to remove any organic compounds that could have disturbed the EDS analyses. 

Then the EDS analyses were performed with an AV of 15.00 kV, a magnification of 204×, and a working distance of 8.5 mm to compare the tested exposed healing screws with the unexposed ones (previously named as group E and group F). 

## 3. Results

### 3.1. Microbial Procedure 

The culture-dependent techniques showed a positive growth of the incubated salivary bacteria in the enriched broth medium. The anaerobic colonies enumerated from the sonicated solution of the healing screws were different between the groups but remained low in all of them, as shown in Table 1. In particular, groups B and D, which were exposed to the application of 14% doxycycline gel, showed a lower CFU count than the control groups (Table 1). 

Regarding the identification of the anaerobic strains, *Prevotella melaninogenica* was the most prevalent together with the different species of facultative anaerobes such as *Streptococcus salivarius*. However, among the detected species, those more associated with periodontitis and peri-implantitis were *Fusobacterium periodonticum* and *Streptococcus mitis*, which were not present in the groups treated with the doxycycline gel (Table 2). The microbial diversity shown in the different groups also indicated a species selection that may be derived from the different surfaces and the different exposures to the antibiotic gel.

### 3.2. SEM-EDS Analyses 

The SEM observation in SE mode at different magnifications showed the presence of the bacterial colonies on the groups where the antibiotic gel was not applied and the active presence of the gel in the group tested. 

**Figure 1:**
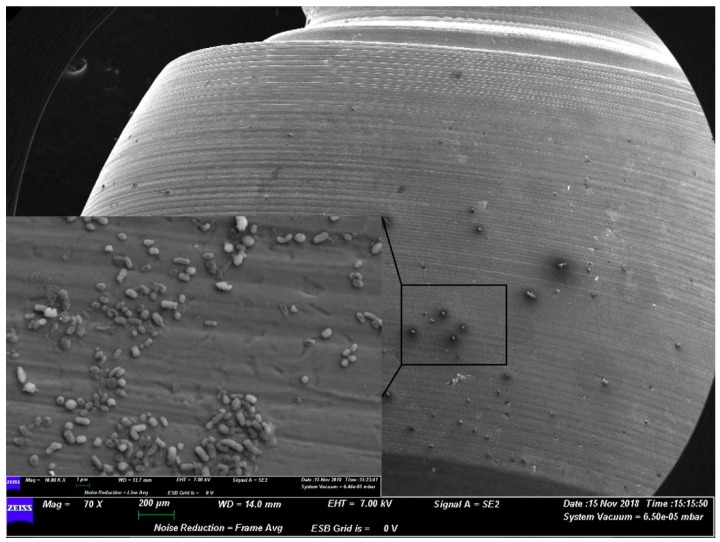
Representative SEM observation of a surface from group A. The microorganisms appear morphologically healthy.

Indeed, the morphology of the microorganisms appeared as healthy and sane colonies on the surface belonging to group A (i.e., the anatase surface with the bacteriostatic action) (Figure 1). 

**Figure 2:**
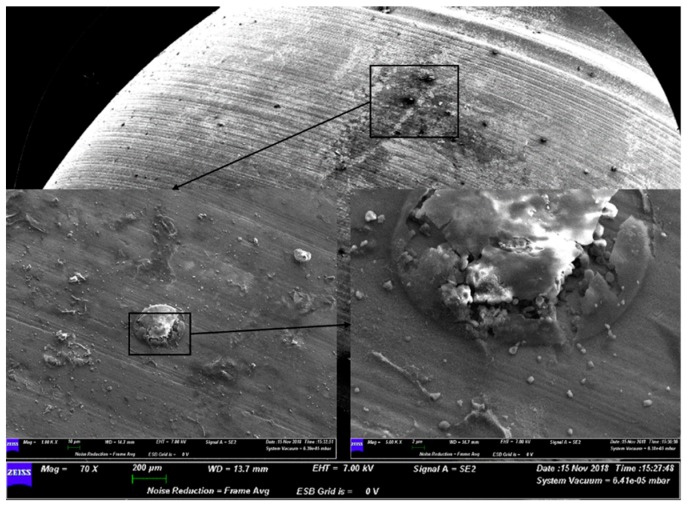
Representative SEM observation of a surface from group B. The magnifications show the action of the gel on the bacterial colonies.

Instead, on the surface of group B, the SEM images showed the active action of the doxycycline on the colonies. Microorganisms did not appear rounded or exhibit a preserved shape (Figure 2). 

**Figure 3:**
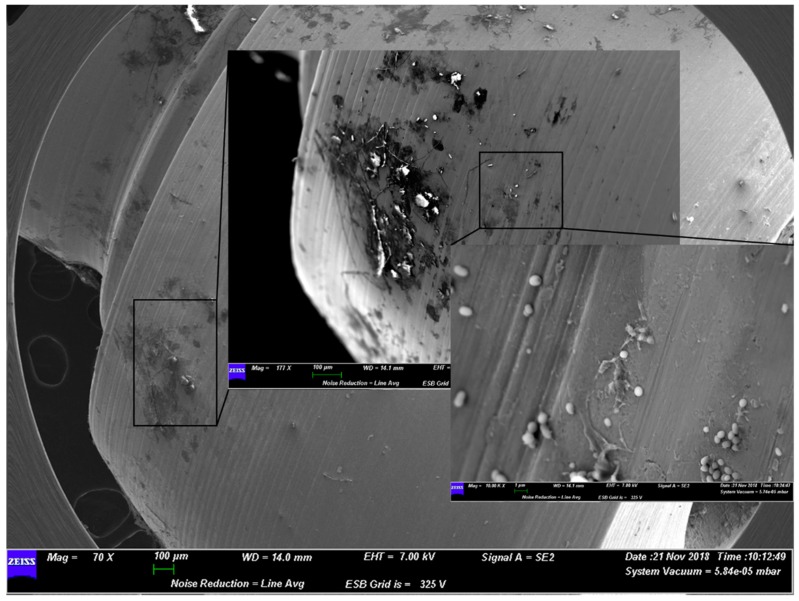
Representative SEM observation of a surface from group C. The microorganisms appear morphologically healthy with a well-preserved shape.

**Figure 4:**
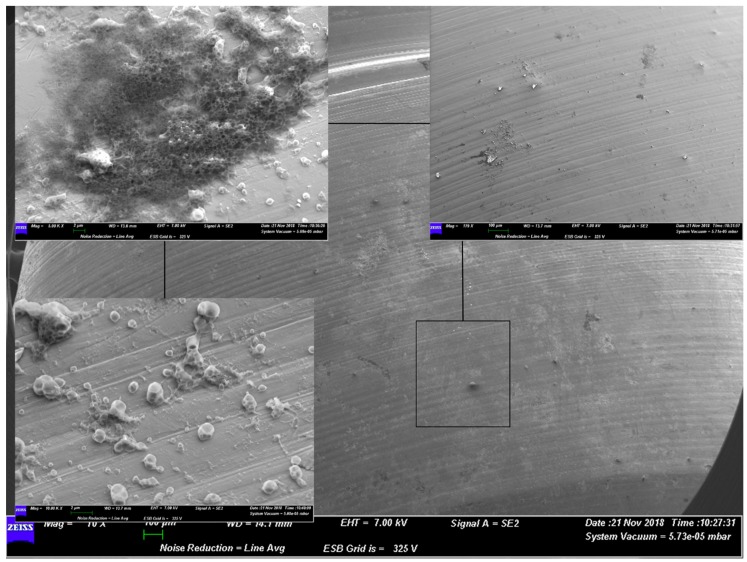
Representative SEM observation of a surface from group D. The magnifications show the action of the gel on the bacterial colonies. The external appearance of microorganisms does not show a well-preserved shape of the cells.

A similar situation was present for group C and group D. The former, which used grade 5 titanium alloy, presented a well-structured layer of biofilm on the surface. The latter instead showed how the gel acted on the bacterial shape (Figure 3 and Figure 4). 

**Figure 5:**
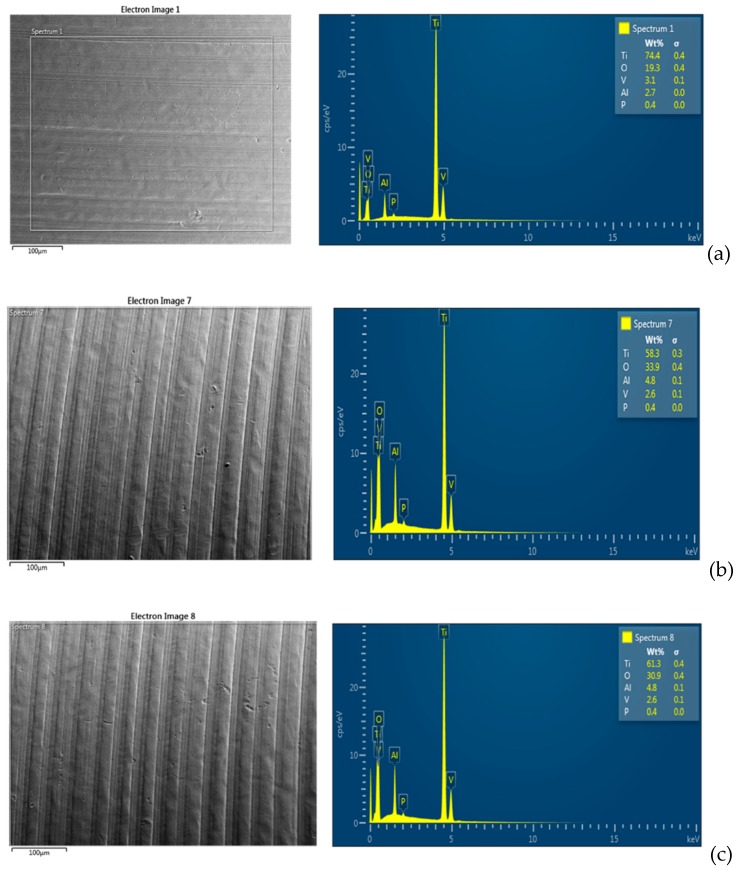
SEM-energy dispersive X-ray spectrometry (EDS) representative spectrum. (**a**) Analysis of group E. (**b**) Analysis of the surfaces in group A and (**c**) analysis of the surfaces in group B. There is no particular difference between the groups exposed to saliva with and without the gel application. Instead, there is a small difference between the surfaces of the exposed groups and the unexposed groups. In particular, there is an increase of oxygen (O) and aluminum (Al), and a small decrease of titanium (Ti) and vanadium (V) on the surfaces of the exposed groups.

**Figure 6:**
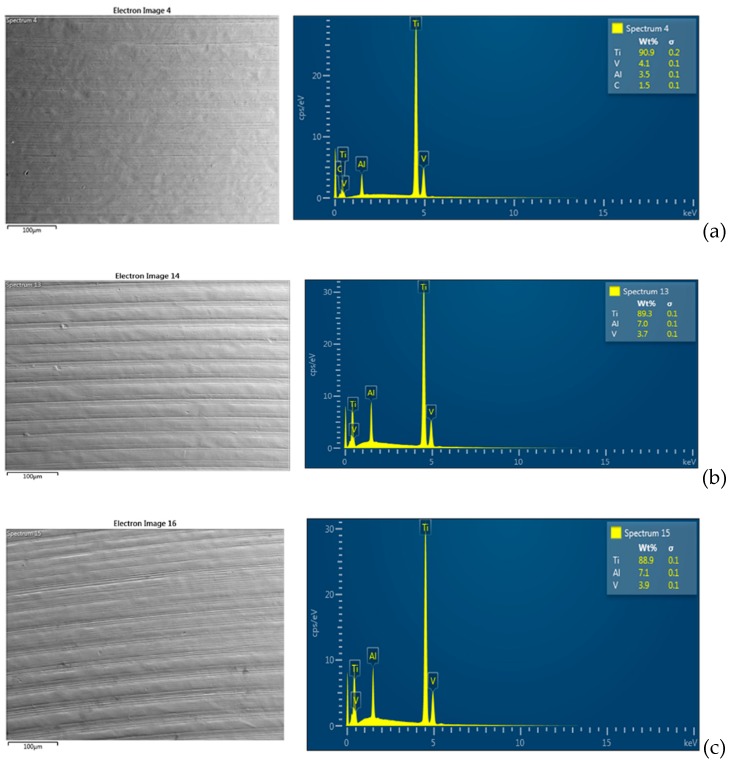
SEM-EDS representative spectrum. (**a**). Analysis of group F. (**b**) Analysis of the surfaces in group C and (**c**) analysis of the surfaces in group D. There is no particular difference between the groups exposed to saliva with and without the gel application. Instead, there is a small difference between the surfaces of the exposed groups and the unexposed groups. In particular, there is an increase of aluminum (Al) and a small decrease of titanium (Ti) and vanadium (V) on the surfaces of the exposed groups.

Regarding the SEM-EDS analyses, the observations were performed at three different random points on the surface of each sample. As shown in Figure 5 and Figure 6, compared to the negative control groups (groups E and F), there was a decrease of the Ti element for all of the groups exposed to microbial adhesion (groups A, B, C, and D). 

There was no difference between the surface exposed to doxycycline and the surface exposed only to microorganisms. It is likely that the minimal decrease of the element Ti is due to the metabolisms of the microorganisms and the preparation methods of the samples for SEM-EDS observation. 

## 4. Discussion

The oral environment has a very peculiar ecological system, where microorganisms living within biofilms co-exist with the tissues in the oral cavity [22,23]. Due to its anatomical structure and functions, its sterility is impossible, and therefore every chemical and mechanical strategy to control the microbial load of the oral environment is crucial to maintaining good oral health. In this context, the strategies for keeping the environment healthy and for facing any biofilm-mediated disease involving the surface of an implant are widely studied [13,24].

The core problem of infections mediated by a biofilm covering an abiotic surface is due to the protective role towards the microorganisms [22].

Therefore, in case of peri-implantitis, the strategy for preventing and facing this infectious oral pathology includes not only the administration of oral antiseptic or topical antibiotic, but also the development of surfaces that are easy to mechanically clean and can be made as bacteriostatic as possible [6].

Therefore, when applied, local antimicrobial molecules must be effective towards the bacterial population; at the same time, they should not have any harmful effect on the titanium surface.

For example, as recently reported by Fukushima et al., sodium fluoride, a molecule that is very important for caries prevention, can induce titanium corrosion in an acidic pH [25].

Also, the same biofilm in the determined condition can lead to the modification of the titanium oxide layer due to the microbial metabolism [25].

The tested topical antibiotic (Ligosan^®^, Heraeus Kulzer GmbH, Hanau, Germany) is composed of two main components: 14% doxycycline and a carrier consisting of a hydrogel polymer that allows the release of the molecule and that degrades in glycolic acid and lactic acid. For years, the efficacy of this product as an adjunctive and promising strategy to treat periodontitis has been studied [26]. Indeed, the bacterial strains that decrease after the use of 14% doxycycline gel in patients suffering from periodontal disease are *Aggregatibacter actinomycetemcomitans*, *Tannerella forsythia*, *Porphyromonas gingivalis*, and *Treponema denticola* [26]. However, its use to also treat peri-implantitis has been considered by the scientific community only for a few years.

Beyond the evaluation of its antibiotic activity, which has been assessed only on specific strains, the eventual effect of the gel on the surface together with human saliva had not been considered prior to this study.

Our results confirmed the active action of doxycycline on anaerobic strains that are typical of chronic periodontitis. Indeed, the microbial growth was in poor agreement with the other few studies present in the literature. As reported by Ferreira et al., tetracycline paste is efficient in reducing the contamination of *Escherichia coli* and Porphyromonas gingivalis on implant sand-blasted acid-etched surfaces [27].

Patianna et al. showed that the same product tested in our study is effective on Streptococcus sanguinis, the microorganism fundamental to the beginning of biofilm formation [19].

Patianna et al. also highlighted the other influencing factor in biofilm formation: The topography of the titanium surface, of which the degree of roughness did not influence biofilm formation [19].

The role played by the degree of roughness of the implant surface is still an open debate. Indeed, the literature widely reports studies correlating the roughness of the surface with microbial adhesion, and a rough surface does not always have more microbial adhesion compared to a smooth surface [28]. These results may be due to the binding reaction between the cellular appendices such as fimbriae and pili and the type of roughness of the surface [29]. In our study, to exclude the parameter of the degree of roughness, a smooth titanium surface was used.

However, the possible action of the degradation product of the polymer carrying the antibiotic has not been previously evaluated.

Indeed, one of these products, lactic acid, was previously investigated by Qu et al., who investigated its action on a titanium surface immersed in artificial saliva. They found that in that condition, lactic acid accelerates the corrosion of the superficial layer of TiO_2_ [30].

In our study, where human saliva was used, the comparison between the groups where the gel was applied and the groups exposed to human saliva but without gel application showed no difference.

Relevant information derived from the SEM observations was the high permanence of the gel on the surface, even though the samples were washed and immersed in an ascending series of alcohols.

Since, according the manufacturer’s instructions for the treatment of periodontitis, the gel has to remain on the surface for 12 days, and the product degrades after 50 days, a final mechanical polishing of the surface at the last follow-up after two months would be recommended. Indeed, polymer degradation could serve as a substratum for new biofilm formation.

Prospective randomized clinical trials are highly recommended to establish the best strategy to use topical antibiotic application to address peri-implantitis.

Limitations of this study include the relatively small size of the considered surfaces and the consequent lack of an appropriate statistical analysis. A future in vitro study considering different concentrations of the saliva sample is suggested to deeply assess the antimicrobial effectiveness of 14% doxycycline gel on titanium surfaces. Another limitation is the source of the saliva. Indeed, a more appropriate source of salivary microorganisms would have been gingival sulcus saliva, due to the difference of the microbial population in different areas of the oral cavity. As reported by Simon Soro et al. [31], the regions of the oral cavity can harbor and create different micro-environments with the selection of several species. In addition, since the same study assessed diversity of the microbial biofilm composition even in the different surfaces of the same tooth, the establishment of a sampling protocol of the microbial oral biofilm is definitely needed for in future in vivo studies assessing the efficacy of a topical antibiotic in the case of peri-implantitis.

## 5. Conclusions

The topical antibiotic strategy is one of several available therapies used to address peri-implantitis. Its use on titanium together with human saliva in anaerobic conditions does not affect the superficial structure of the surface.

## Figures and Tables

**Table 1:** Colony-forming units (CFU) count of the different tested groups.

Group	CFU/mL
A	3 × 10^−3^
B	2 × 10^−3^
C	3 × 10^−3^
D	1.4 × 10^−3^

**Table 2:** Species retrieved in the different groups.

Group A	Group B	Group C	Group D
*Prevotella melaninogenica*	*Prevotella melaninogenica*	*Prevotella melaninogenica*	*Prevotella melaninoenica*
*Streptococcus mitis*	*Streptococcus parasanguis*	*Prevotella buccae*	*Streptococcus cristatus*
*Fusobacterium periodonticum*	*Actinomyces odontoliticus*	*Streptococcus oralis*	*Streptococcus oralis*
*Streptococcus vestibularis*	*Streptococcus vestibularis*	*Veillonella dispar*	
	*Streptococcus salivarius*	*Streptococcus salivarius*	
